# Supplementary material for: The causal relationship between the human gut microbiota and pyogenic arthritis: a Mendelian randomization study
Source: Front Cell Infect Microbiol. 2024 Nov 26;14:1452480. doi: 10.3389/fcimb.2024.1452480 (PMC11629706; doi:10.3389/fcimb.2024.1452480)
Supplement: Supplementary file 1 [file DataSheet1.zip › mendelian test/ebi-a-GCST90027792.csv_forest.pdf]

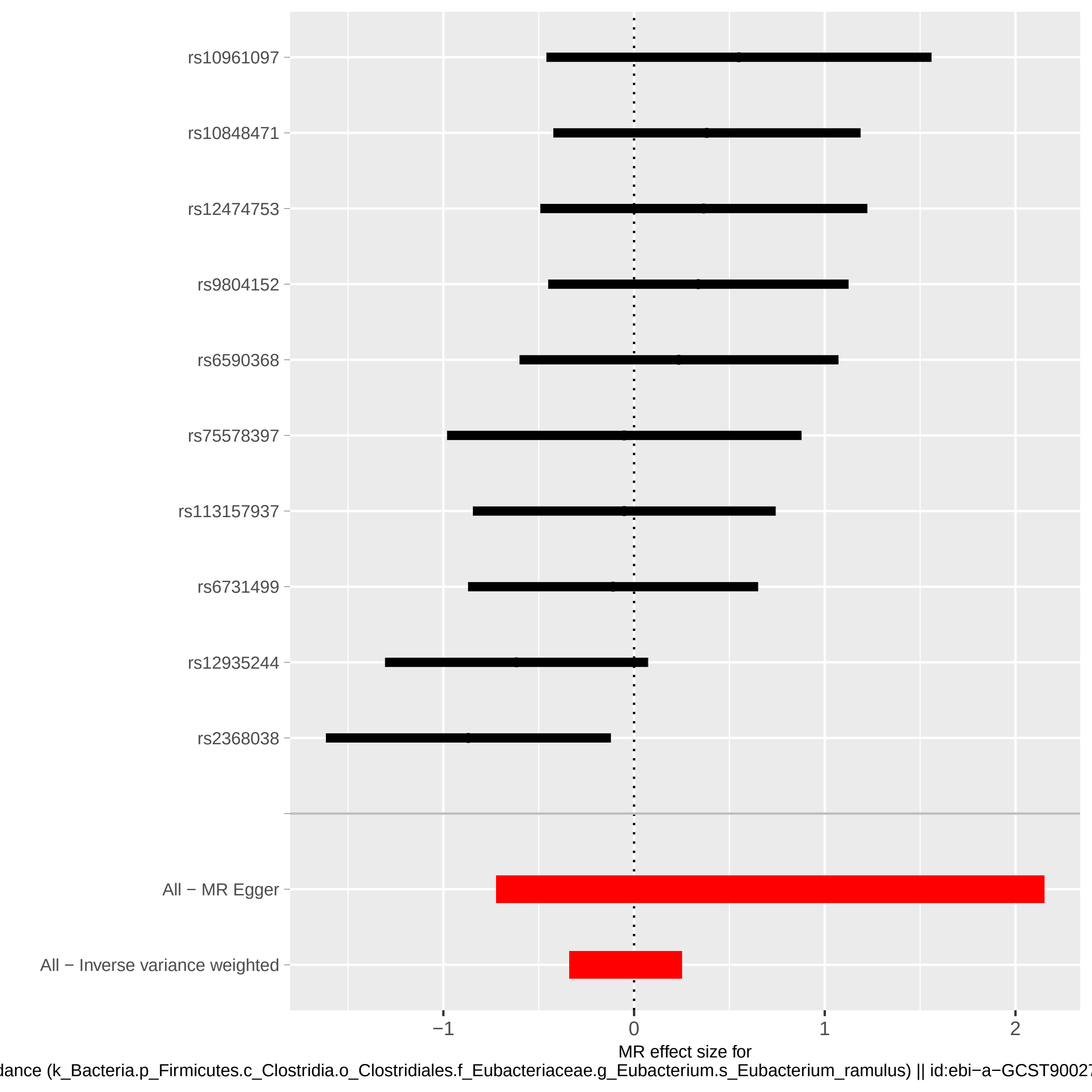

rs10961097
rs10848471
rs12474753
rs9804152
rs6590368
rs75578397
rs113157937
rs6731499
rs12935244
rs2368038
All − MR Egger
All − Inverse variance weighted
−1
0
1
2
MR effect size for
dance (k_Bacteria.p_Firmicutes.c_Clostridia.o_Clostridiales.f_Eubacteriaceae.g_Eubacterium.s_Eubacterium_ramulus) || id:ebi−a−GCST9002
